# Draft Genome Sequence of the Sordariomycete *Lecythophora* (*Coniochaeta*) *hoffmannii* CBS 245.38

**DOI:** 10.1128/genomeA.01510-17

**Published:** 2018-02-15

**Authors:** Sabrina Leonhardt, Enrico Büttner, Anna Maria Gebauer, Martin Hofrichter, Harald Kellner

**Affiliations:** a Department of Bio- and Environmental Sciences, International Institute Zittau, Technische Universität Dresden, Zittau, Germany

## Abstract

*Lecythophora* (*Coniochaeta*) *hoffmannii*, a soil- and lignocellulose-inhabiting sordariomycete (Ascomycota) that can also live as a facultative tree pathogen causing soft rot, belongs to the family Coniochaetaceae. The strain CBS 245.38 sequenced here was assembled into 869 contigs, has a size of 30.8 Mb, and comprises 10,596 predicted protein-coding genes.

## GENOME ANNOUNCEMENT

*Lecythophora* (*Coniochaeta*) *hoffmannii* (J. F. H. Beyma) Gams and McGinnis (1983) is a filamentous ascomycetous mold that belongs to the family Coniochaetaceae (order Sordariales). Here, we present the second genome sequence of this family (1). The fungus is a facultative plant pathogen, colonizes as saprotroph soils, leaf litter, and coarse wood debris, and can cause opportunistic mycosis in humans (2–4). Ecophysiologically, *L. hoffmannii* is a soft-rot fungus and is able to utilize aromatic compounds (phenolics and aryl alcohols/aldehydes) that emerge during wood decomposition (2). In particular, the fungus colonizes the surface of wood, the lignified cell walls of which it penetrates with thin hyphae. The process of wood decomposition is mediated mainly by extracellular glycosidases (5, 6), such as cellulases and xylanases (7–9). Analysis of 28S rRNA genes of the genus *Lecythophora* revealed that the taxon appears as a monophyletic group related to teleomorphs of the genus *Coniochaeta*; however, for the species sequenced here, no teleomorph is currently known (10). Since *L. hoffmannii* is widespread and has strong activity on lignocelluloses, its genome provides a significant resource for biotechnological and ecological studies.

*L. hoffmannii* CBS 245.38 (GenBank accession number MG491499) was obtained from the Westerdijk Fungal Biodiversity Institute (CBS, Utrecht, Netherlands). The strain was cultivated in 2.5% malt extract medium under submerged conditions, and after harvest, the genomic DNA was extracted using a cetyltrimethylammonium bromide (CTAB) protocol. The DNA was sonographically fragmented using an S2 system (Covaris, Woburn, MA, USA) to generate a 200-bp fragment library (Ion Plus fragment library kit; Thermo Fisher Scientific, Darmstadt, Germany). Then, the DNA was sequenced using an Ion Torrent PGM system and the Ion PGM Sequencing 200 kit (version 2, 318v2 chip). The assembly of 5.5 million reads was performed using MIRA 4.0 (11) (which resulted in 1,770 contigs) and a second assembly step using the Geneious R10 assembler (12) filtered for duplicate contigs. A final assembly consisting of 869 contigs with a length of 30.8 Mb was generated. Genome single-copy ortholog analysis performed with BUSCO (Benchmarking Universal Single-Copy Orthologs) software (predictor *Aspergillus nidulans*) (13) reported a genome completeness of 91.5%. Quality statistics were analyzed using QUAST (Quality Assessment Tool for Genome Assemblies) version 4.5 (14), and we evaluated an *N*_50_ of 59,573 bp and an average G+C content of 55.8%. Using the AUGUSTUS Web server (species parameter, *Aspergillus nidulans*), 10,596 protein-coding genes were predicted (15). Enzyme-encoding genes related to wood decomposition were annotated and filtered using Blast2GO (BioBam, Valencia, Spain). Analysis of genes using dbCAN (16) and the CAZy database identified 556 enzymes, which are categorized into 262 glycoside hydrolases, 99 carbohydrate esterases, 9 polysaccharide lyases, 90 glycosyltransferases, 96 enzymes with auxiliary (oxidative) activities, and 73 carbohydrate-binding modules (CBM) (17 from CBM family 1 binding to cellulose). The following enzymes may be directly involved in the degradation of wood polysaccharides: 9 endo-1,4-β-xylanases (4 GH11 and 5 GH10), 15 endo-1,4-β-glucanases (5 GH5 and 10 GH7), and 2 cellobiose dehydrogenases. Peroxidase genes were also assessed; however, merely one nonligninolytic, generic, class II-related peroxidase was found. On the other hand, 2 long unspecific peroxygenases (UPOs) belonging to the heme-thiolate proteins (17) and 10 laccases were identified (GenBank accession numbers MG550044 to MG550081).

### Accession number(s).

This whole-genome shotgun project has been deposited at DDBJ/ENA/GenBank under the accession number NXFW00000000. The version described in this paper is version NXFW01000000.

## References

[1] Jiménez DJ, Hector RE, Riley R, Lipzen A, Kuo RC, Amirebrahimi M, Barry KW, Grigoriev IV, van Elsas JD, Nichols NN 2017 Draft genome sequence of *Coniochaeta ligniaria* NRRL 30616, a lignocellulolytic fungus for bioabatement of inhibitors in plant biomass hydrolysates. Genome Announc 5:e01476-16. doi:10.1128/genomeA.01476-16. 28126934 PMC5270693

[2] Bugos RC, Sutherland JB, Adler JH 1988 Phenolic compound utilization by the soft rot fungus *Lecythophora hoffmannii*. Appl Environ Microbiol 54:1882–1885. 16347701 10.1128/aem.54.7.1882-1885.1988 PMC202766

[3] Marriott DJE, Wong KH, Aznar E, Harkness JL, Cooper DA, Muir D 1997 *Scytalidium dimidiatum* and *Lecythophora hoffmannii*: unusual causes of fungal infections in a patient with AIDS. J Clin Microbiol 35:2949–2952. 9350765 10.1128/jcm.35.11.2949-2952.1997 PMC230093

[4] Khan Z, Gené J, Ahmad S, Cano J, Al-Sweih N, Joseph L, Chandy R, Guarro J 2013 *Coniochaeta polymorpha*, a new species from endotracheal aspirate of a preterm neonate, and transfer of *Lecythophora* species to *Coniochaeta*. Antonie Van Leeuwenhoek 104:243–252. doi:10.1007/s10482-013-9943-z. 23748934

[5] Hale MD, Eaton RA 1985 The ultrastructure of soft rot fungi. II. Cavity-forming hyphae in wood cell walls. Mycologia 77:594–605. doi:10.2307/3793358.

[6] Sigoillot J-C, Berrin J-C, Bey M, Lesage-Meessen L, Levasseur A, Lomascolo A, Record E, Uzan-Boukhris E 2012 Fungal strategies for lignin degradation, p 263–308. *In* Jouanin L, Lapierre C (ed) Advances in botanical research, vol 61 Academic Press, Burlington, VT.

[7] Nilsson T 1973 Studies on wood degradation and cellulolytic activity of microfungi. Studia Forestalia Suec 104:1–40.

[8] Nilsson T 1974 The degradation of cellulose and the production of cellulase, xylanase, mannanase and amylase by wood-attacking microfungi. Studia Forestalia Suec 114:1–61.

[9] Nilsson T 1974 Microscopic studies on the degradation of cellophane and various cellulosic fibres by wood-attacking microfungi. Studia Forestalia Suec 117:1–32.

[10] Weber E, Görke C, Begerow D 2002 The *Lecythophora-Coniochaeta* complex II. Molecular studies based on sequences of the large subunit of ribosomal DNA. Nova Hedwigia 74:1–2, 187–200.

[11] Chevreux B, Wetter T, Suhai S 1999 Genome sequence assembly using trace signals and additional sequence information. *In* Computer science and biology, Proceedings of the German Conference on Bioinformatics (GCB) 99, p 45–56.

[12] Kearse M, Moir R, Wilson A, Stones-Havas S, Cheung M, Sturrock S, Buxton S, Cooper A, Markowitz S, Duran C, Thierer T, Ashton B, Meintjes P, Drummond A 2012 Geneious Basic: an integrated and extendable desktop software platform for the organization and analysis of sequence data. Bioinformatics 28:1647–1649. doi:10.1093/bioinformatics/bts199. 22543367 PMC3371832

[13] Simão FA, Waterhouse RM, Ioannidis P, Kriventseva EV, Zdobnov EM 2015 BUSCO: assessing genome assembly and annotation completeness with single-copy orthologs. Bioinformatics 31:3210–3212. doi:10.1093/bioinformatics/btv351. 26059717

[14] Gurevich A, Saveliev V, Vyahhi N, Tesler G 2013 QUAST: quality assessment tool for genome assemblies. Bioinformatics 29:1072–1075. doi:10.1093/bioinformatics/btt086. 23422339 PMC3624806

[15] Stanke M, Steinkamp R, Waack S, Morgenstern B 2004 AUGUSTUS: a Web server for gene finding in eukaryotes. Nucleic Acids Res 32:W309–W312. doi:10.1093/nar/gkh379. 15215400 PMC441517

[16] Yin Y, Mao X, Yang JC, Chen X, Mao F, Xu Y 2012 dbCAN: a Web resource for automated carbohydrate-active enzyme annotation. Nucleic Acids Res 40:W445–W451. doi:10.1093/nar/gks479. 22645317 PMC3394287

[17] Hofrichter M, Kellner H, Pecyna MJ, Ullrich R 2015 Fungal unspecific peroxygenases: heme-thiolate proteins that combine peroxidase and cytochrome P450 properties. Adv Exp Med Biol 851:341–368. doi:10.1007/978-3-319-16009-2_13. 26002742

